# Quantification of Intramyocardial Metabolites by Proton Magnetic Resonance Spectroscopy

**DOI:** 10.3389/fcvm.2015.00024

**Published:** 2015-05-12

**Authors:** Lisa Gillinder, Shi Yi Goo, Gary Cowin, Mark Strudwick, Rob J. van der Geest, William Y. S. Wang, Arnold C. T. Ng

**Affiliations:** ^1^Department of Cardiology, Princess Alexandra Hospital, The University of Queensland, Brisbane, QLD, Australia; ^2^Centre for Advanced Imaging, The University of Queensland, Brisbane, QLD, Australia; ^3^Department of Radiology, Leiden University Medical Center, Leiden, Netherlands

**Keywords:** magnetic resonance, spectroscopy, myocardial, metabolites, left ventricle

## Abstract

**Purpose:**

To define intramyocardial triglyceride (TG), creatine (Cr), and choline (Cho) in healthy volunteers, and determine the feasibilities, scan durations and agreements between cardiac proton magnetic resonance spectroscopy ([^1^H]-MRS) performed with fewer signal averages versus a reference standard with 128 signal averages.

**Materials and methods:**

Thirty-one participants underwent [^1^H]-MRS using 16, 32, 64, and 128 signal averages. Intramyocardial TG, Cr, or Cho contents relative to water were calculated and expressed as a percentage.

**Results:**

Mean intramyocardial TG, Cr, and Cho were 1.30 ± 1.13, 0.19 ± 0.18, and 0.24 ± 0.28%, respectively. The feasibilities for quantifying intramyocardial TG, Cr, and Cho using fewer signal averages ranged from 93.5 to 100, 90.3 to 93.5, and 90.3 to 96.8%, respectively. Scan durations for 16, 32, 64, and 128 signal averages were 1.1 ± 0.5, 2.6 ± 0.9, 5.9 ± 2.0, and 13.2 ± 4.5 min, respectively (*p* < 0.001). Agreements with the reference standard 128 signal average was higher for quantification of intramyocardial TG compared to Cr and Cho.

**Conclusion:**

Quantification of intramyocardial TG with [^1^H]-MRS with only 64 signal averages was highly feasible, showed excellent agreement with 128 signal averages, and had significantly shorter scan duration. By contrast, quantifying Cr and Cho using fewer signal averages had lower feasibilities and agreements compared to 128 signal averages.

## Introduction

**Figure 1:**
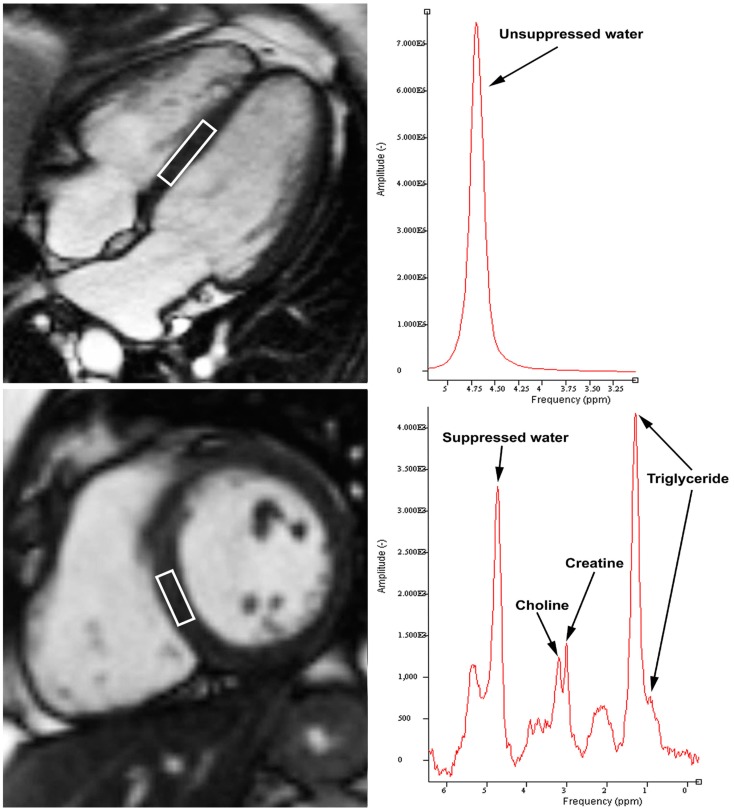
**Example of cardiac proton magnetic resonance spectroscopy with voxel of interest placed at interventricular septum (left panels)**. Unsuppressed water content peaks at 4.7 ppm (top right panel). Intramyocardial triglyceride was quantified by summing the amplitudes of lipid resonances at 0.9 and 1.3 ppm, while choline and creatine peaks at 3.2 and 3.0 ppm, respectively (bottom right panel).

The study of intracellular metabolites in response to pathophysiological stimuli can provide insights into the mechanisms underlying disease states. Currently, one of the methods used for *in vivo* quantification of these metabolites includes proton magnetic resonance spectroscopy ([^1^H]-MRS) (Figure 1) using point resolved spectroscopy sequence (PRESS) with cardiac and respiratory gating (1). Previous [^1^H]-MRS studies have demonstrated changes in intramyocardial concentrations of triglyceride (TG), creatine (Cr), and choline (Cho) in various cardiac pathologies such as diabetic heart disease and heart failure (2–7). Intramyocardial TG is increased in diabetic and obese patients in a process synonymous with “fatty liver” disease (2–4). Intramyocardial Cr is a marker of the total cardiac energetic system and is required in the production of adenosine triphosphate (ATP). Creatine depletion has been shown to correlate well with the extent of myocardial ischemia post infarction and the degree of myocardial dysfunction in cardiac failure (8–10). Choline has a wide role in human metabolism, including neurotransmitter synthesis (acetylcholine), cell-membrane signaling (phospholipids), lipid transport (lipoproteins), and methyl-group metabolism (homocysteine reduction). Although previous studies have evaluated intramyocardial TG in healthy and diabetic patients (2–4), no studies to date have quantified intramyocardial Cr and Cho in a cohort of healthy volunteers.

Presently, quantification of intramyocardial metabolites by [^1^H]-MRS is frequently performed using 32 up to 128 signal averages (Figure 1) (11–13). Acquiring more signal averages will improve spectral quality by improving signal-to-noise ratio. However, acquiring 128 signal averages is extremely time consuming due to need for cardiac and respiratory motion gating to reduce cardiac and respiratory motion artifacts (11). Often, patients cannot tolerate extended periods of examinations within the magnetic resonance imaging (MRI) machine. No studies to date have evaluated the agreements between [^1^H]-MRS of different signal averages.

Therefore, the aims of the present study were to quantify intramyocardial TG, Cr, and Cho in a cohort of healthy volunteers, and to determine the feasibilities, scan durations, and agreements between [^1^H]-MRS performed with fewer signal averages versus the reference standard with 128 signal averages.

## Materials and Methods

### Patient population and study protocol

A total of 31 healthy volunteers without known medical illnesses were prospectively recruited. These subjects were specifically recruited in this research study to determine intramyocardial metabolite levels in healthy people. Exclusion criteria included age <18 years, pregnancy, rhythm other than sinus rhythm, previously known significant coronary artery disease, left ventricular (LV) systolic dysfunction or previous myocardial infarction, moderate or severe valvular stenosis or regurgitation, diabetes mellitus, hypercholesterolemia, active smoker, inability to provide informed consent, and inability to undergo a cardiac MRI.

All participants underwent a cardiac MRI examination after an overnight fast. LV volumes, ejection fraction (EF), and mass were quantified. Intramyocardial metabolites (including TG, Cr, and Cho) were quantified by [^1^H]-MRS. The feasibilities, accuracies, and scan durations of individual separate water-suppressed [^1^H]-MRS signals with only 16, 32, and 64 averages were compared with the reference standard spectrum with 128 signal averages.

The study was approved by the institutional ethics committee and all subjects provided informed consent.

### Cardiac magnetic resonance imaging

All participants underwent a cardiac MRI examination using a 1.5-T Siemens Magnetom Avanto (Erlangen, Germany) system. During the examination, the entire heart was imaged in the short-axis orientation with ECG-gated breath-hold balanced steady state free-precession imaging. Imaging parameters included the following: echo time (TE) = 1.0 ms, repetition time (TR) = 57.9 ms, flip-angle = 54°, slice thickness = 8 mm with a gap of 2 mm, field of view = 340 mm × 340 mm, reconstructed matrix size = 156 × 192.

Left ventricular end-diastolic volume index (EDVI) and end-systolic volume index (ESVI) were measured and corrected for body-surface area (BSA) (14). LVEF was then calculated and expressed as a percentage. LV mass (excluding papillary muscles) was also measured and indexed to BSA. All images were digitally stored on hard disks and analyzed offline using dedicated quantitative software (MASS V2010-EXP, Leiden University Medical Center, Leiden, The Netherlands).

### Cardiac proton MR spectroscopy

Cardiac [^1^H]-MRS was performed as previously described (2). Briefly, [^1^H]-MRS spectra were obtained by PRESS with a 6 mL voxel (1 cm × 2 cm × 3 cm) placed in the interventricular septum using the four-chamber and short-axis views at end-diastole. Spectroscopic data acquisitions were double-triggered with ECG triggering and respiratory navigator echoes to minimize motion artifacts. End-diastolic spectra were acquired with the following parameters: TE = 25 ms, TR = 2000 ms, 1024 data points, bandwidth 1000 Hz. Automatic shimming was performed before the spectroscopy data acquisition using the gradient-recalled echo shim technique whereby a field map was generated from a single-slab double-echo 3D gradient-recalled echo acquisition with two in-phase TEs with respect to fat and water, which were then used to calculate the shim currents to improve B0 homogeneity.

Water-suppressed spectra were acquired to quantify intramyocardial metabolites (including TG, Cr, and Cho). To determine the feasibilities, scan durations, and accuracies of water-suppressed [^1^H]-MRS with fewer signal averages, individual spectra with 16, 32, and 64 signal averages were acquired and compared with the reference standard spectrum with 128 signal averages. Non-water-suppressed spectra were acquired with 16 signal averages and used as an internal standard (see next section). In between spectra acquisitions, the volume of interest was kept fixed within the interventricular septum and not adjusted.

### Spectral quantification

[^1^H]-MRS data were fitted by use of Java-based MR user interface software (jMRUI version 2.2, Leuven, Belgium) AMARES algorithm as previously described (2). Water-suppressed spectra with 16, 32, 64, and 128 averages were used to quantify intramyocardial TG, Cr, and Cho content. Resonance frequency estimates for intramyocardial TG, Cr, and Cho were described by assuming Lorentzian line shapes. Intramyocardial TG was quantified by summing the amplitudes of lipid resonances at 0.9 and 1.3 ppm, while Cho and Cr peaks at 3.2 and 3.0 ppm, respectively (Figure 1). Quantification of intramyocardial TG, Cr, and Cho was deemed not feasible if no clear peaks were visible at the expected respective resonance frequencies, and when the Lorentzian curve fitting by jMRUI could not be performed. To determine the adequacy and accuracy of the fitted signal amplitude, the relative Cramer–Rao standard deviation (CRSD) was calculated as previously published (15). The CRSD was determined using the AMARES algorithm, and then divided by the lipid signal amplitude. This generates a relative CRSD, which is inversely proportional to signal-to-noise ratio.

Unsuppressed spectra were acquired with 16 averages to determine water content, which peaks at 4.7 ppm. This water peak signal was used for internal standardization, and its quantification was feasible in all subjects.

Intramyocardial TG, Cr, and Cho contents relative to water were calculated and expressed as a percentage based on: (signal amplitude of TG, Cr, or Cho)/(signal amplitude of water) × 100 (2).

### Statistical analysis

Continuous variables were presented as mean ± 1 SD unless otherwise stated, and categorical variables were presented as frequencies and percentages. Comparisons between the two groups of subjects with feasible versus any non-feasible [^1^H]-MRS analyses were performed using Mann–Whitney *U*-test and Chi-square test for continuous and categorical variables, respectively. Pearson correlation was employed to examine the linear association between two continuous variables. Comparison of feasible versus any non-feasible [^1^H]-MRS analyses with increasing number of signal averages (i.e., ordinal data) was performed using Chi-square test with linear-by-linear association. Agreements between [^1^H]-MRS of fewer signals averages (i.e., with 16, 32, and 64 signal averages) and the reference standard spectrum with 128 signal averages were performed using intraclass correlations and Bland and Altman plots (16). Differences between the four groups of [^1^H]-MRS of different signal averages were compared using repeated measures analysis of variance (ANOVA). A two-tailed *p* value of <0.05 was considered significant. All statistical analyses were performed using IBM SPSS Statistics for Windows, version 21.0 (Armonk, NY, USA).

## Results

A total of 31 participants (21 male) were recruited. The mean age and BMI were 35 ± 11 years and 24.4 ± 4.4 kg/m^2^, respectively. The mean height was 175.2 ± 9.1 cm and weight was 75 ± 14 kg. The mean heart rate, LVEDVI, LVESVI, LVEF, and LV mass were 66 ± 12 beats/min, 92.6 ± 15.9 mL/m^2^, 41.8 ± 7.6 mL/m^2^, 54.5 ± 3.0%, and 86.5 ± 22.6 g, respectively.

### Intramyocardial metabolites in healthy volunteers

As shown in Table 1, using 128 signal averages as the reference standard, it was possible to quantify intramyocardial TG and Cho in all 100% of patients, and intramyocardial Cr in 96.8% of patients. The mean intramyocardial TG, Cr, and Cho concentrations were 1.30 ± 1.13, 0.19 ± 0.18, and 0.24 ± 0.28%, respectively. There were no significant differences in intramyocardial TG, Cr, and Cho between male and female gender (all *p* > 0.05). Similarly, there were no correlations between intramyocardial TG, Cr, and Cho with age, LV mass, volumes, and EF (all *p* > 0.05).

**Table 1:** **Intramyocardial metabolite contents and intraclass correlations for spectra with fewer signal averages versus reference standard with 128 signal averages**.

Number of signals	Triglyceride (ICC)	Creatine (ICC)	Choline (ICC)
16 averages	1.34 ± 1.22% (0.941)	0.27 ± 0.30% (0.585)	0.27 ± 0.37% (0.872)
32 averages	1.33 ± 1.18% (0.973)	0.21 ± 0.21% (0.650)	0.24 ± 0.25% (0.915)
64 averages	1.43 ± 1.27% (0.977)	0.22 ± 0.20% (0.812)	0.24 ± 0.27% (0.924)
128 averages (reference standard)	1.30 ± 1.13%	0.19 ± 0.18%	0.24 ± 0.28%

*ICC, intraclass correlation*.

### Scan durations and feasibilities of spectroscopy with fewer signals

**Figure 2:**
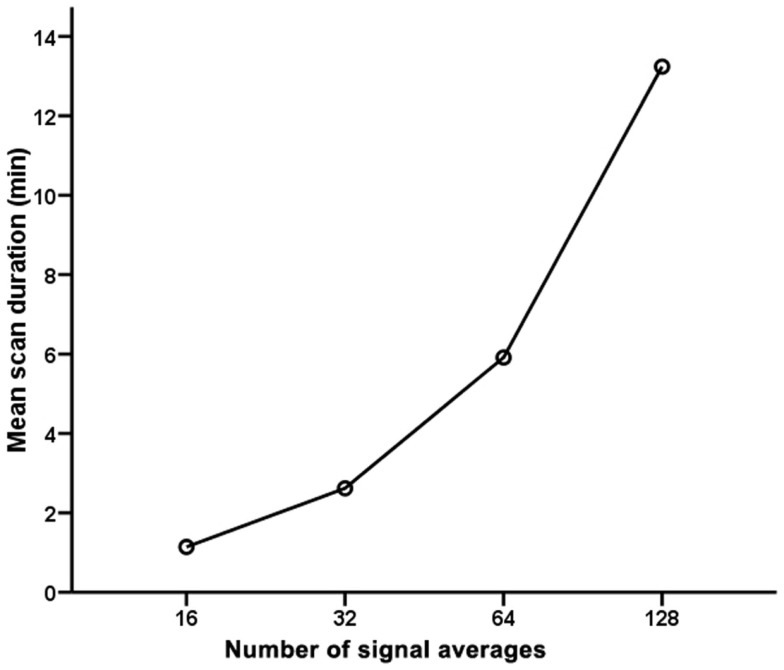
**Scan durations for cardiac proton magnetic resonance spectroscopy with 16, 32, 64, and 128 signal averages**.

Table 2 outlines the scan durations and feasibilities for quantifying intramyocardial TG, Cr, and Cho contents relative to water. The mean scan duration for the reference standard [^1^H]-MRS with 128 signal averages was 13.2 ± 4.5 min (range 10.1–29.7 min). As expected, [^1^H]-MRS with fewer signal averages had significantly shorter scan durations compared to the reference standard (*p* < 0.001 by repeated measures ANOVA; Figure 2).

**Table 2:** **Scan durations and feasibilities of cardiac proton magnetic resonance spectroscopy for quantifying intramyocardial metabolites**.

Number of signals	Scan duration (min)	Triglyceride (% feasibility)	Creatine (% feasibility)	Choline (% feasibility)
16 averages	1.1 ± 0.5	93.5	90.3	90.3
32 averages	2.6 ± 0.9	100	90.3	96.8
64 averages	5.9 ± 2.0	100	93.5	96.8
128 averages (reference standard)	13.2 ± 4.5	100	96.8	100

**Figure 3:**
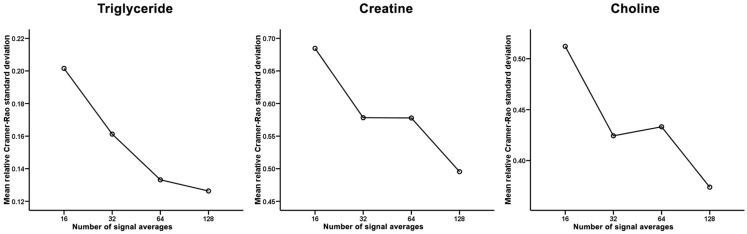
**Mean relative Cramer–Rao standard deviation for the intramyocardial triglyceride, creatine, and choline**. Increasing signal averages resulted in progressively lower relative Cramer–Rao standard deviation and therefore a higher signal-to-noise ratio.

However, [^1^H]-MRS with fewer signal averages had lower feasibilities for quantification of intramyocardial metabolites (Table 2). Accordingly, the total feasibilities for quantifying all 3 intramyocardial metabolites using [^1^H]-MRS of 16, 32, 64, and 128 signal averages were 77.4, 87.1, 90.3, and 96.8%, respectively (*p* = 0.020 by Chi square with linear-by-linear association). As a result, it was possible to quantify all 3 intramyocardial metabolites from all 4 spectra of different signal averages in 23 subjects. Figure 3 shows the mean relative CRSD (an inverse measure of the signal-to-noise ratio of the accuracy of the fitted signal amplitude) for the intramyocardial metabolites. As expected, increasing signal averages were associated with an improvement in the signal-to-noise ratio.

Compared to the 23 subjects where it was possible to quantify all 3 intramyocardial metabolites from all 4 spectra, participants with any non-feasible [^1^H]-MRS analyses had a trend toward lower intramyocardial TG (0.92 ± 0.90 versus 1.43 ± 1.19%, *p* = 0.30), Cr (0.17 ± 0.16 versus 0.19 ± 0.19%, *p* = 0.68), and Cho contents (0.10 ± 0.06 versus 0.29 ± 0.31%, *p* = 0.26). There were no significant differences in age, gender, heart rate, LV mass, volumes, or EF (all *p* > 0.05).

### Agreements with reference standard spectroscopy

**Figure 4:**
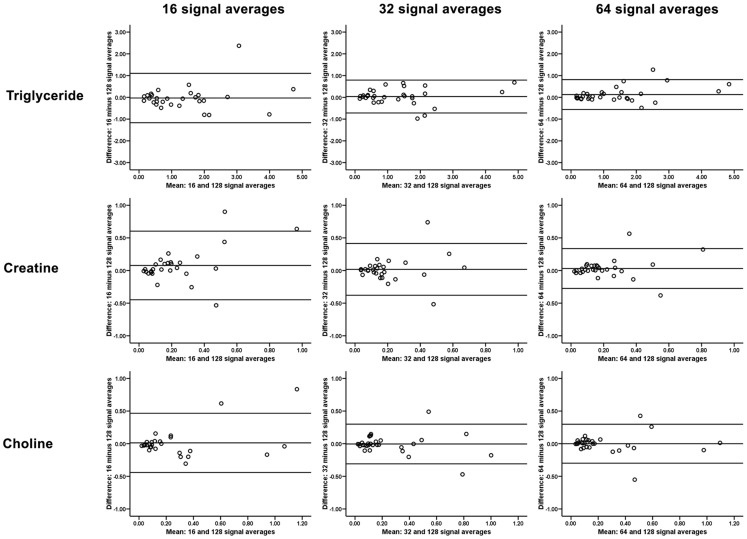
**Bland and Altman plots showing narrower limits of agreement and no significant bias for quantification of intramyocardial triglyceride, creatine, and choline with progressively more signal averages acquired during proton magnetic resonance spectroscopy**.

Table 1 outlines the intramyocardial TG, Cr, and Cho contents using [^1^H]-MRS of different number of signal averages, and their intraclass correlations with the reference standard [^1^H]-MRS with 128 signal averages. As shown, [^1^H]-MRS with more signal averages had better agreements with the 128 signal average reference standard, and Bland and Altman plots showed progressively narrower limits of agreement with no significant bias (Figure 4). As a group, there were no significant differences in intramyocardial TG, Cr, and Cho contents derived from [^1^H]-MRS of differing signal averages (all *p* > 0.05 by repeated measures ANOVA).

## Discussion

The present study evaluated intramyocardial TG, Cr, and Cho in a large cohort of healthy volunteers, and demonstrated that it was feasible to quantify intramyocardial metabolites using fewer [^1^H]-MRS signal averages and shorter scan duration. However, the accuracies and signal-to-noise ratios were suboptimal when intramyocardial metabolite concentrations were low, especially for Cr and Cho compared to TG. Therefore, intramyocardial TG can be accurately quantified with significantly lower signal averages once a discernible peak is visible on [^1^H]-MRS, but accurate quantification of Cr and Cho should be performed using 128 signal averages as the reference standard.

### Physiological roles of intramyocardial metabolites

Quantification of intramyocardial metabolites can provide insights into various disease states. A typical myocardial [^1^H]-MRS spectrum will display signals from water, TG, Cr, and Cho as shown in Figure 1. Under normal physiological conditions, the heart utilizes both free fatty acids and glucose as the primary source of energy. However, increased nutritional fatty acid intake and increased lipolysis in diabetes or obesity lead to increased free fatty acid delivery to the heart (17–19). The excessive fatty acids are stored as TGs within the myocyte cytoplasm, and part of it is redirected into non-oxidative pathways giving rise to toxic fatty acid intermediates (5, 20) These toxic fatty acid intermediates disrupt normal cellular signaling and alter myocyte function and structure, eventually causing cellular apoptosis and replacement fibrosis. Previous study has demonstrated that intramyocardial TG content increases with normal aging, and is inversely correlated with age-related decline in LV diastolic function (3). Similarly, intramyocardial TG accumulation occurs in pathological states such as diabetes, and is dependently associated with myocardial dysfunction (21–23). These findings were replicated in experimental animal studies showing a direct toxic effect of fatty acid intermediates on the myocardium (24, 25). Therefore, it is likely that intracellular TG is inert, but is reflective of the increased intracellular concentrations of toxic fatty acid intermediates.

In contrast, Cr is a marker of cardiac energetic system and is required in the production of ATP in the Krebs cycle (5). [^1^H]-MRS can quantify the total pool of phosphorylated and unphosphorylated creatine in the myocardium, and is more sensitive than phosphorus MRS (26). Both Bottomley et al. and Nakae et al. previously demonstrated that the total pool of Cr is reduced in patients with ischemic heart disease and heart failure, respectively (9, 26). Furthermore, total Cr pool was significantly lower in patients with more severe heart failure (9).

Finally, Cho has a wide ranging role in human metabolism and is required for neurotransmitter synthesis (acetylcholine), cell-membrane signaling (phospholipids), lipid transport (lipoproteins), and methyl-group metabolism (homocysteine reduction) (6). Dietary deficiency of Cho may be associated with myocardial fibrosis and steatosis, restrictive pericarditis, myocardial necrosis, pericardial effusions, and atherosclerosis (6, 7). However, there is limited literature on quantification of intramyocardial Cho concentration.

### Cardiac proton magnetic resonance spectroscopy

There are multiple fundamental technical challenges, which have significantly limited the use of [^1^H]-MRS despite considerable clinical potential (27). Currently, quantification of intramyocardial metabolites by [^1^H]-MRS is performed using 128 signal averages as a clinical reference standard (11). However, acquiring 128 signal averages is time consuming due to need for cardiac and respiratory motion gating to reduce motion artifacts. In the present study, the scan durations for acquiring [^1^H]-MRS with 128 signal averages ranged from approximately 10 to 30 min, and were not inclusive of the time required for scan planning and other clinical routine sequences such as cine. Therefore, total MRI examination duration is usually longer than 1 h when performing [^1^H]-MRS. To the best of our knowledge, the present study is, first, to determine the feasibilities, scan durations, and accuracies of performing [^1^H]-MRS with fewer signal averages. For intramyocardial TG that has higher relative concentrations compared to Cr and Cho, [^1^H]-MRS can be performed with significantly fewer signal averages and still maintain high technical feasibility without loss of quantification accuracy. It could be expected that clinical studies of patients with diseased myocardium would be even more feasible using fewer averages, as intramyocardial TG levels are increased. However, acquiring fewer signal averages decreases the signal-to-noise ratio as reflected in the higher CRSD. Therefore, intramyocardial metabolites such as Cr and Cho, which have significantly lower relative concentrations, could not be easily and accurately quantified using fewer signal averages. This would be compounded in clinical scenarios where patients with heart failure or myocardial infarction will have metabolite depletion (10). Furthermore, the clear spectral separation of the Cr and Cho peaks at 3.0 and 3.2 ppm, respectively, is reduced when fewer [^1^H]-MRS signals were acquired. It is also less feasible to accurately quantify all three intramyocardial metabolites in patients with inherently low concentrations without acquiring 128 averages.

## Conclusion

In summary, quantification of intramyocardial TG with [^1^H]-MRS can be accurately performed using only 64 averages with significant time savings. Therefore, it is feasible to use 64 averages to quantify intramyocardial TG in future clinical studies. However, quantification of Cr and Cho should be performed using the more sensitive clinical reference standard of 128 signal averages due to their low intramyocardial concentrations.

## Conflict of Interest Statement

Associate Professor Arnold C. T. Ng is supported by the NHMRC early career fellowship. The study was supported by an unrestricted educational grant by Abbott Australasia Pty Ltd.
